# Radiation Characterization of Smart Aerogels Based on Hollow VO_2_ Particles

**DOI:** 10.3390/gels11040273

**Published:** 2025-04-05

**Authors:** Xingcan Li, Shengkai Qin, Bowei Xie, Tianbo Hou, Linkang Wang, Yinmo Xie, Meiran Han

**Affiliations:** 1 College of Energy and Power Engineering, Northeast Electric Power University, Jilin 132012, China; 2 Institute for Advanced Technology, Shandong University, Jinan 250061, China; 3 Shenzhen Research Institute of Shandong University, Shandong University, Shenzhen 518000, China; 4 Shandong Key Laboratory of Thermal Science and Smart Energy Systems, Shandong University, Jinan 250061, China; 5 Department of Mechanical and Electrical Engineering, Jiangxi Water Resources Institute, Nanchang 330013, China; 6 School of New Energy, Harbin Institute of Technology, Weihai 264209, China; 7 College of International Chinese Studies, Beijing Language and Culture University, Beijing 100083, China

**Keywords:** aerogel, hollow particles, VO_2_, T-matrix method, Monte Carlo method, emittance, modulation

## Abstract

The smart aerogel control technology based on thermochromic materials can dynamically adjust the emittance with temperature changes, which plays a significant role in reducing energy consumption and carbon emissions. This paper presents the design of the smart aerogel based on hollow VO_2_ particles with excellent emittance modulation. The radiation characteristics of a single particle were calculated using the multi-sphere superposition T-matrix method, and the radiation characteristics of the aerogel were determined using the Monte Carlo method. The results indicate that when the radius of the hollow VO_2_ particles is 1 μm and the shell thickness is 40 nm, the hollow particles display excellent thermal regulation. When the thickness of the VO_2_ particle smart aerogel is 50 μm, with a volume fraction of 2.5%, the emittance of the adaptable aerogel can reach 51.295%, which provides a theoretical foundation for the further advancement of infrared smart aerogels to enhance their energy-saving performance.

## 1. Introduction

As global energy demand grows rapidly and environmental conditions deteriorate, the goal of achieving carbon neutrality by 2060 has gained universal agreement. Energy saving and emission reduction are essential strategies to mitigate carbon emissions and address global warming [1]. Furthermore, immediate action is required to adopt energy-saving and emission-reduction measures [2]. In developed countries, buildings accounted for about 40% of the country’s total energy consumption in 2018, and this share is expected to increase further [3]. Smart thermochromic coatings are anticipated to lower energy consumption by dynamically adjusting outdoor solar radiation, thereby contributing to a reduction in the primary energy usage of buildings. Vanadium dioxide (VO_2_), a typical metal oxide thermochromic material [4], with an intricately complex electronic phase diagram and highly tunable orbital configurations, demonstrates the sharpest reversible phase transition from the metallic to the insulating phase at a phase change temperature of 68 °C (341 K), near room temperature [5,6]. Its resistance varies by up to 5.0% before and after the phase transition. Its electrical resistance varies by up to five orders of magnitude before and after the phase transition. Simultaneously, the optical constants of VO_2_ in the infrared region increase significantly before and after the thermochromic phase transition [7], which can take into account both light and energy saving, and thus has a potential advantage in regulating the internal temperature of the building to realize the “warmth in winter and coolness in summer” [8,9]. Hence, VO_2_ is considered one of the most promising materials for smart aerogels. However, key challenges remain in practical applications, including the high phase transition temperature (with the human body’s comfort temperature being approximately 25 °C), reduced light transmittance, and limited light modulation capabilities of VO_2_. Scholars have explored various strategies in order to improve the transmittance and light modulation properties of VO_2_ [10]. Huang et al. [11] proposed a scalable adaptive radiative cooling membrane. By introducing VO_2_-based core–shell nanoparticles into a polyethylene matrix, a radiation-cooling membrane with adaptive capability was obtained. This study provides a promising solution for scalable radiatively cooled films with high adaptive cooling performance and durability. Improvements were made through doping, transmittance enhancement design, and stacking of composites [12].

To theoretically simulate the film formation of VO_2_ nano smart particles, Li et al. [13] constructed an optical model for the distribution of VO_2_ nanoparticles in a homogeneous and transparent matrix based on the effective medium theory. The effective medium theory (Bruggeman) involves the calculation of new values of optical constants n and k for two materials of core–shell particles by means of the following formula. The conclusion of their simulation proved that the films with VO_2_ nanoparticles dispersed in the matrix showed excellent performance in terms of light transmittance as well as light modulation ability compared with the VO_2_ films. Thin films with dispersed core–shell particles have a Tsol difference of 20% between the semiconductor and metal phases, compared to a Tsol difference of only 7% for a single film, with a 13% improvement in tunability. Cao et al. [14] chose an inexpensive and environmentally friendly H_2_O layer as a movable reflective reduction layer. Seasonal indoor/outdoor temperature difference changes caused the adaptive dynamic modulation of the gas–liquid two-phase of the H_2_O layer, which was able to significantly improve the optical properties of VO_2_ films. This method achieves 42.5% T_lum_ and 18.2% ∆T_sol_, breaking the bottleneck of utilizing magnetron sputtering to obtain large ∆T_sol_. Xu et al. [15] proposed an ARC/VO_2_ bilayer structure, which was used in the 100–300 °C range. The refractive index of ARC was adjusted to 1.69 in the annealing temperature range to provide higher anti-reflective properties at low temperatures than at high temperatures, thus maximizing the thermal modulation of different kinds of VO_2_ nanolayers. Xie et al. [16] proposed a wavelength-selective emitter that has been designed to achieve multiband stealth and dual-band heat dissipation. The emitter demonstrates low emittance of 0.159, 0.057, and 0.067 in the short-wave, mid-wave, and long-wave infrared bands, respectively, and exhibits high absorption of 0.811 in the visible to near-infrared bands.

Zheng et al. [17] designed and prepared large-area, multifunctional TiO_2_(A)/VO_2_(M)/TiO_2_(R) multilayers integrating energy-saving, anti-fogging, and self-cleaning functions. The bottom TiO_2_(R) layer has a lattice constant similar to that of VO_2_(M), which improves the crystallinity of VO_2_ and also acts as an anti-fog reduction layer to achieve energy saving. Yang et al. [18] introduced an all-season smart film that employs phase change materials (VO_2_ and IST) to achieve a synergistic modulation of solar and thermal radiation. The optimal solar absorptance in the range of 0.3~2.5 μm and thermal emittance in the 8~13 μm range for the solar heating mode and radiative cooling mode are (a_normal_, ε_normal_) = (0.706, 0.093) and (0.300, 0.952).

Liu et al. [19] dispersed VO_2_ nanoparticles with a particle size of close to 20 nm, which were obtained by the ball milling method, into a transparent Si-Al-based gel to form thermochromic films. In this process, Si and Al migrated to the surface of VO_2_ nanoparticles and formed a shell-like structure wrapped around the VO_2_ nanoparticles, compared with nanoporous films; with slightly reduced D_Tsol_ (12% vs. 14.1%), dramatically enhanced averaged T_lum_ (59% vs. 41.6%) can be achieved.

Xie et al. [20] proposed a VO_2_-based spacecraft smart radiator with low solar absorptance and a simple structure with highly enhanced infrared emittance and emittance tunability that outperforms the existing thermochromic films; the irradiance tunability of the stacked FP films for thermochromic films can exceed 0.70, while the total normal irradiance at elevated temperatures is greater than 0.91. The maximum total normal emissivity of the stacked FP films can be as high as 0.95, with an emissivity tunability of 0.78.

Zhao et al. [21] proposed a visible and transparent thermal insulating film to reduce energy loss from windows. By embedding insulator–metal phase transition vanadium dioxide (VO_2_) nanoparticles in an ultra-low thermal conductivity aerogel film, the film is transparent in visible light and can be dynamically switched in solar energy transmission. Chen et al. [22] proposed to synthesize a composite porous aerogel utilizing chitosan-derived carbon aerogel as a scaffold loaded with VO_2_ (VO_2_/CA), which exhibits excellent radar/infrared-compatible stealth properties.

In 1931, Kistler pioneered the production of silica aerogel through the supercritical drying method, employing water glass as the primary raw material and ethanol as the drying solvent. This milestone signaled the commencement of research endeavors into aerogel materials. Aerogel, a distinctive material, is characterized by its remarkably low density, exceptional void structure, and an exceedingly high specific surface area. It finds extensive applications in construction, aerospace, and numerous other fields. Klemmed et al. [23] pioneered the exploration of a hybrid equipartitioned excitatory element aerogel material doped with gold nanorods. This material is engineered by modulating the length-to-diameter ratio of the nanorods to stimulate resonance peaks at specific wavelengths, while concurrently enhancing the absorption at the corresponding wavelengths. Berquist et al. [24] demonstrated that the infrared equipartitioned excitation element aerogel material can be utilized as a thermal conductor material by incorporating the infrared equipartitioned excitation element indium tin oxide (ISTO) into silica aerogel. Zhu et al. [25] proposed a core–shell structured light shielding agent particles and explored the effects of core–shell ratio, particle size, and core–shell material on the radiative transfer within the core–shell structured light shielding agent-doped composite silica aerogel.

The theoretical and computational study of VO_2_ micro–nano structures has advanced significantly, with much of the research focused on the cladding structures of hollow VO_2_ particles. However, studies on hollow particle structures remain relatively limited. This work calculates the radiation characteristics of hollow VO_2_ particles by altering their spatial structure while employing BaF_2_ crystals as the aerogel substrate, which are renowned for their excellent optical and mechanical properties. Hollow VO_2_ particles are randomly doped into BaF_2_ crystal aerogels, and solar transmittance is regulated through VO_2_’s reversible phase transition, enabling intelligent control of infrared emittance in response to external conditions. By investigating the effects of different layer thicknesses and volume fractions on the spectral properties of the smart aerogel, this study lays a theoretical foundation for optimizing smart aerogels to improve energy savings.

## 2. Results and Discussion

### 2.1. Validation of the Method

**Figure 1:**
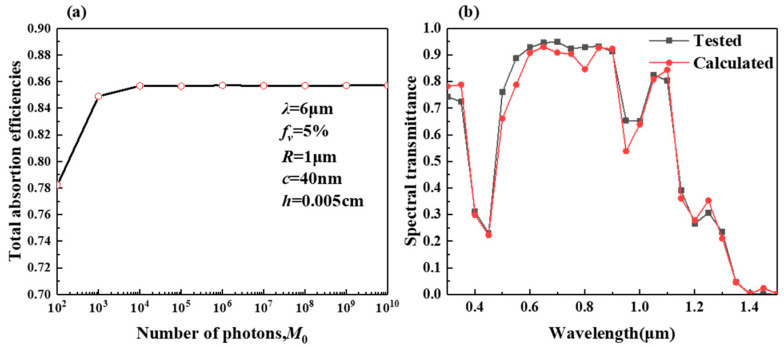
(**a**) Verification of the determination of the number of photons and (**b**) Monte Carlo calculations and experimental validation.

As the number of photons increases, as shown in Figure 1a, the absorption curve of the simulation calculation becomes more stable, and the calculation results are accurate with enough number of photons. Therefore, in this work, the number of photons calculated by simulation is 10^8^. To validate the feasibility of the Monte Carlo method developed in this study, the spectral transmittances of Ag/water nanofluids with varying mass fractions, as suggested by prior studies, were utilized. During the validation process, the average radius of the silver nanoparticles was set to 25 nm, and the refractive index of the surrounding medium was set at 1.33. Figure 1b depicts the calculated directional–directional spectral transmittances for 10 mm optical thickness by the Monte Carlo method and compares them with the experimental results from reference [2]. The average relative error is 3.2264%.

### 2.2. Radiation Properties of the Hollow VO_2_ Particles

#### 2.2.1. Effect of Different Layer Thicknesses c on the Particle Absorption Factor for a Particle Size of *r*_2_ = 1 μm

**Figure 2:**
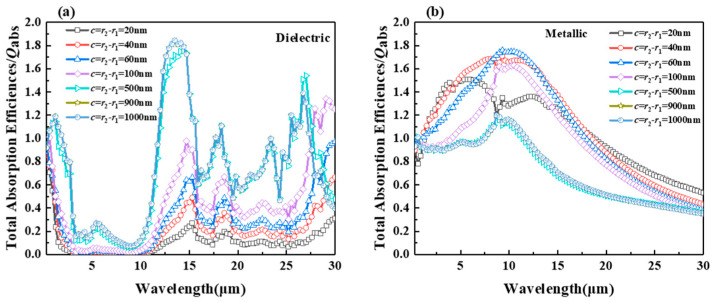
(**a**) The absorption factor for different shell thicknesses c in the dielectric state of VO_2_ and (**b**) the absorption factor for different shell thicknesses c in the metallic state of VO_2_.

Figure 2 shows the absorption factor of the hollow VO_2_ particles in the (a) dielectric and (b) metallic state with different shell thicknesses and *r*_2_ = 1 μm. As shown in Figure 2a, the absorption factor of hollow VO_2_ particles in 0.3–3 μm decreases with the increase in wavelength, shows a broad trough in 3–12.5 μm and a sharp peak in 15 μm, and then oscillates dramatically. The absorption factor of the core–shell particles for the dielectric VO_2_ state shows a tendency to increase with the increase in the thickness of the VO_2_ shell. When the thickness of the hollow VO_2_ particles’ layer is 40 nm, its absorption factor is the smallest throughout the thickness for the wavelength. The peak near the wavelength of 15 μm appears to be blue-shifted. In the spectral range of 15–30 μm, the absorption factor of the particles also shows a strong oscillation with the increase in VO_2_ thickness. With the increase in VO_2_ shell thickness up to 900 nm, the absorption factor of the particles is almost independent of the shell thickness. According to Figure 2a, the largest change in the particle absorption factor occurs in the spectral range of 15–30 μm, depending on the change in the thickness of the VO_2_ shell. The broader absorption troughs and lower absorption factor values observed for VO_2_ shell thicknesses between 20 and 100 nm are primarily attributed to the combined effects of the core–shell cavity and the optical properties of the VO_2_ material. In this range, the cavity effect of the particles tends to have a favorable effect on changing the absorption factor of the particles. It is evident that the absorption factor of solid spherical particles (shell thickness: 1000 nm) remains largely unaltered when compared with that of particles with a shell thickness of 900 nm.

As can be seen from Figure 2b, the absorption coefficient of hollow VO_2_ particles increases with the wavelength in the 0.3–5 μm spectral range, peaks in the 5–12.5 μm range, and then decreases as the wavelength increases. In the 0.3–12.5 μm spectral range, the absorption factor of the core–shell particles initially increases and then decreases as the shell thickness of the hollow VO_2_ particles increases. Near the 10 μm spectral wavelength, the absorption factor of the core–shell particles reaches its maximum at a shell thickness of 60 nm, although the peak is not the broadest. At a shell thickness of 20 nm for VO_2_ particles, two fluctuations in the absorption factor are observed within the 5–10 μm range, and these fluctuations increase with shell thickness. As can be seen in Figure 2b, a strong and wide peak of the absorption factor appears as the shell thickness of the hollow VO_2_ particles varies in the range of 20–100 nm. The reason for this phenomenon is the cavity effect within the core–shell particles and the result of the material together. In summary, the difference between the metallic and dielectric states of hollow VO_2_ particles with a shell thickness of 20–100 nm remains around 1.5 in the spectral range of wavelengths 3–15 μm. It is concluded that the thickness of the particle shell of hollow VO_2_ particles at 40 nm has the most superior difference between the metallic and dielectric states.

#### 2.2.2. Effect of Different Radii on the Absorption Factor of Nucleoshell Particles at VO_2_ Shell Thickness of 40 nm

**Figure 3:**
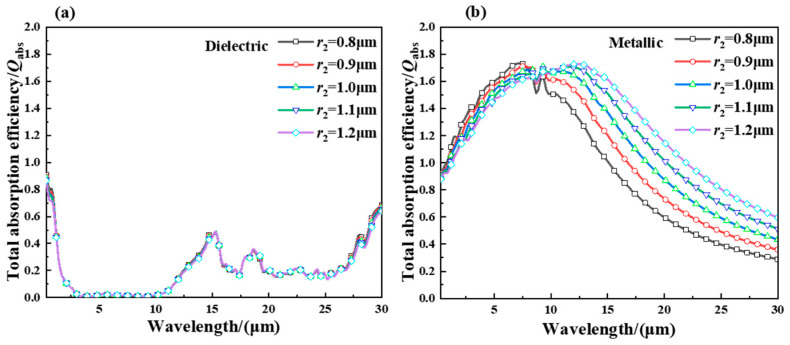
(**a**) Absorption efficiency for different radii of the dielectric state of VO_2_ and (**b**) absorption efficiency for different radii of the metallic state of VO_2_.

Figure 3a shows the absorption factor of the hollow VO_2_ particles in the (a) dielectric and (b) metallic state with a different radius and shell thickness of 40 nm. It can be concluded from Figure 3a that the absorption factor of the hollow particles with the dielectric VO_2_ state is almost independent of the radius of the particles. As can be seen from Figure 3b, the absorption factor for metallic VO_2_ in the range of spectral wavelength 0.3–7.5 μm increases with the increase in radius of hollow particles; in the range of spectral wavelength 10–30 μm, the absorption factor of hollow particles decreases with the increase in particle radius. At wavelengths of 0.3–7.5 μm, the absorption factor increases with increasing wavelength, and at wavelengths of 10–30 μm, the absorption factor decreases with increasing wavelength. The reason for this is that at wavelengths of 7.5–10 μm, there is an absorption peak in the absorption factor and the phenomenon of redshift with increasing radius, and the size of the cavity of the particles has a significant effect on the absorption peak. It can be analyzed that the absorption factor of hollow particles has a wide bandwidth when the radius is 1 μm.

As can be seen from Figure 3a,b, in the spectral range of wavelengths from 3.0 to 12.5 μm, the difference in absorption factors between the dielectric and metallic states of the hollow particles is more than 1.2. Through comprehensive analysis, the hollow particles exhibit strong self-regulation of the absorption factor when the particle radius is 1 μm and the hollow thickness is 40 nm, with an adjustable value exceeding 1.3, and reaching up to approximately 1.8. This is primarily due to the cavity structure of the hollow particles, which significantly enhances the absorption factor in the metallic state of VO_2_ while having a negligible effect on the absorption factor in the dielectric state.

### 2.3. Spectral Emittance of the Hollow VO_2_ Particles-Based Smart Aerogel

**Figure 4:**
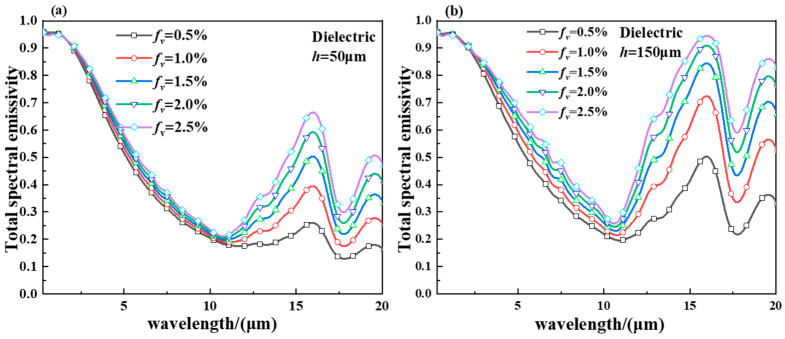
(**a**) Spectral emittance of a dielectric hollow VO_2_ particle smart aerogel with a thickness of *h* = 50 μm and (**b**) spectral emittance of a dielectric hollow VO_2_ particle smart aerogel with a thickness of *h* = 150 μm.

Figure 4 shows the spectral emissivity of the smart aerogel for the dielectric VO_2_ state with a thickness of (a) *h* = 50 μm and (b) *h* = 150 μm. As shown in Figure 4a, when the aerogel thickness is 50 μm, the spectral emittance of the smart aerogels increases with the increase in volume fraction and shows a trough at spectral wavelengths of 11 μm. At spectral wavelengths of 16.5 μm and 19 μm, the spectral emittance of the smart aerogels exhibits peaks and increases with increasing volume fractions. As shown in Figure 4b, the spectral emittance of the smart aerogels increases with the increase in volume fraction, and the increase in spectral emittance of the smart aerogels will be weakened when the volume fraction increases to 2.5%. In summary, the spectral emittance of the smart aerogels increases with the increase in the volume fraction of the aerogel, and the amount of increase in the spectral emittance of the smart aerogels will become smaller for large volume fractions. At a certain doping volume fraction, the spectral emittance of the smart aerogels increases with the increase in the aerogel thickness and also presents a decrease in the increase of the spectral emittance of the smart aerogels when the aerogel thickness is large.

**Figure 5:**
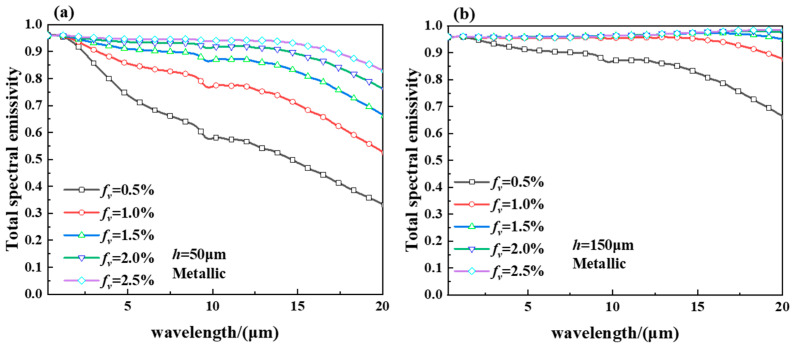
(**a**) Spectral emissivity of a metallic hollow VO_2_ particle smart aerogel with a thickness of *h* = 50 μm and (**b**) spectral emissivity of a metallic hollow VO_2_ particle smart aerogel with a thickness of *h* = 150 μm.

Figure 5 shows the spectral emissivity of the smart aerogel for metallic VO_2_ state with a thickness of (a) *h* = 50 μm and (b) *h* = 150 μm. As can be seen in Figure 5a, when the aerogel thickness is 50 μm, the spectral emittance of the smart aerogel increases with the increase in the volume fraction. As shown in Figure 5b, it can be seen that the spectral emittance of the smart aerogel decreases with the increase in spectral wavelength at 0.5% volume fraction, and the value of the spectral emittance increases with the increase in the volume fraction of the aerogel. The value of spectral emissivity is almost constant with wavelength and shows an increasing trend when the volume fraction is 2.5%. The reason for this is that the volume fraction of the hollow particles increases, and the density of the particles also increases. The scattering intensity between the particles is enhanced accordingly, bringing about an increase in the value of spectral emissivity in the infrared band. When the volume fraction of the smart aerogel is increased to 2.5%, the increase in its spectral emissivity decreases. This is due to the fact that the volume fraction within the aerogel reaches the threshold value, and the effect on the spectral emissivity value is not changing.

### 2.4. Total Emittance of the Hollow VO_2_ Particles-Based Smart Aerogel

**Figure 6:**
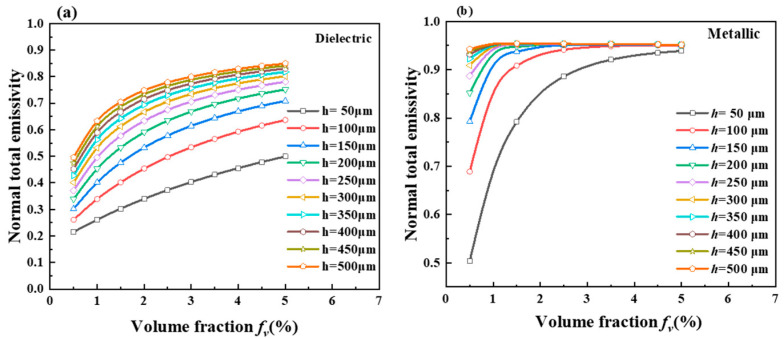
(**a**) Normal total emittance of smart-coated hollow VO_2_ particles in the dielectric state and (**b**) normal total emittance of smart-coated hollow VO_2_ particles in the metallic state.

The smart aerogels exhibit different emittance in metallic and dielectric states after doping with hollow VO_2_ particles. As shown in Figure 6a, the total spectral emissivity of the aerogels generally increases as the volume fraction increases in the dielectric state. When the aerogel thickness is 50 μm, the change in emittance follows a linear trend. In the 100–500 μm range, the change in emittance diminishes considerably with an increase in volume fraction, though it still exhibits an overall increase. At constant volume fractions, the normal total emissivity increases with the aerogel thickness. As can be seen from Figure 6b, at a smart aerogel thickness of 50 μm, the normal total emissivity of the aerogel increases with the increase in the volume fraction. The increment of its emittance is multiplicative in the range of volume fraction 0.5–2.0% for aerogel thickness of 50 μm, then decreases with the increase in the volume fraction in the range of volume fraction 2.0–5.0%, and the value of the normal total emissivity tends to be stabilized gradually. At an aerogel thickness of 100 μm, the normal total emissivity of the smart aerogel increases with the increase in volume fraction, and the normal total emissivity does not increase with the increase in volume fraction after the volume fraction reaches 4.0%. At a smart aerogel thickness of 200–500 μm, the normal total emissivity of the aerogel increases with volume fraction and then remains constant; after the volume fraction reaches 3.0%, the normal total emissivity of the aerogel is not affected by the change in volume fraction. The emittance of the smart aerogels was maintained above 90% at a thickness of 300–500 μm. Based on this phenomenon, it is caused by the fact that the volume fraction of the hollow particles has a great influence on the change in the total normal emittance of the smart aerogels when the aerogel thickness is 50 μm, and the volume fraction has a large influence on the total normal emittance of the smart aerogels. The increase in volume fraction can change the normal total emissivity of the smart aerogel.

**Figure 7:**
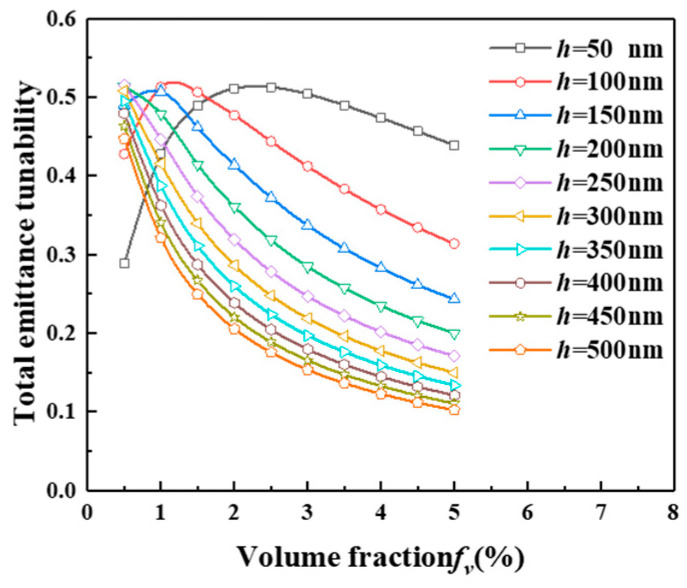
Emittance tunability of the smart aerogels as a function of volume fraction with different aerogel thicknesses.

As can be seen in Figure 7, at a aerogel thickness of 50 μm, the total emittance tunability of the smart aerogels first increases and then decreases with the increase in the volume fraction; at a volume fraction of 2.5%, the total emittance tunability of the smart aerogels reaches the maximum value, which can reach 51.296%. Increasing the volume fraction of hollow particles in the aerogel to 2.5%, the total emittance tunability reaches the threshold of the smart aerogel, and as the volume fraction increases, the concentration of hollow particles will have an inhibitory effect on the total emittance tunability.

## 3. Conclusions

This paper focuses on altering the spatial structure of VO_2_ particles to modify their radiative properties. The radiative properties of hollow VO_2_ particles were computed using the multi-sphere superposition T-matrix method. Additionally, BaF_2_ crystals were employed to create an aerogel substrate with excellent optical and mechanical properties, and hollow VO_2_ particles were randomly incorporated into the BaF_2_ crystal aerogels. The Monte Carlo method was then applied to calculate the emittance and emittance tunability of the smart aerogel. The influence of various layer thicknesses and volume fractions on the spectral properties of BaF_2_-coated films doped with VO_2_ particles was also examined to enhance the optical performance of the VO_2_ aerogels. For individual hollow particles, the absorption factor displays a broad bandwidth when the VO_2_ layer thickness is 40 nm and the spherical particle radius is 1 μm, indicating that the VO_2_ hollow spherical particles have excellent and well-modulated properties.

Theoretical calculations of the radiative transfer properties of the smart aerogels doped with hollow VO_2_ particles show that the total emittance tunability of the aerogels reaches a maximum of 51.295% at an aerogel thickness of 50 μm and a volume fraction of 2.5%. This demonstrates significant optical stability and opens up potential for a wide range of applications of these smart aerogels. As illustrated in Table 1, our work is compared to the existing literature on model design. Our structure is characterized by its simplicity, minimal material usage, enhanced tunability, and an expanded wavelength range [11,26,27,28,29]. Evidently, our research presents a smart thermally controlled aerogel with superior radiative tunability compared to the existing literature. Consequently, it finds application in the domains of aerospace engineering, thermal insulation, and smart window research.

## 4. Materials and Methods

### 4.1. The Models of the Hollow VO_2_ Particle-Based Smart Aerogel

**Figure 8:**
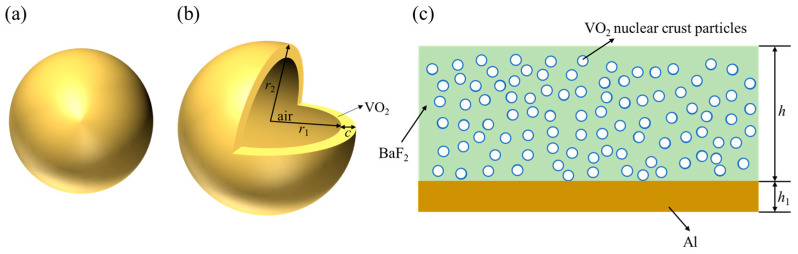
(**a**) Model view of the appearance of hollow VO_2_ particle; (**b**) cross-section of hollow VO_2_ particle; and (**c**) model view of the infrared highly transparent material doped with hollow VO_2_ particles.

Among various research methods to alter the radiation properties of particles, changing the spatial structure of the particles to modify their radiation characteristics is a widely used approach. In this study, the particles are designed as core–shell structures, creating a cavity within the particles. Figure 8 presents the (a) model view and (b) cross-section of the hollow VO_2_ particles. Figure 8c illustrates a schematic diagram of dynamically modulated aerogels formed by the random distribution of hollow VO_2_ particles within a matrix of an infrared highly transparent material. The radiation properties of the particles are then analyzed based on the influence of the cavity structure. Furthermore, the impact of the particles on the radiation transmission properties of smart aerogels is investigated by varying the volume fraction of doped particles and the aerogel thickness.

**Figure 9:**
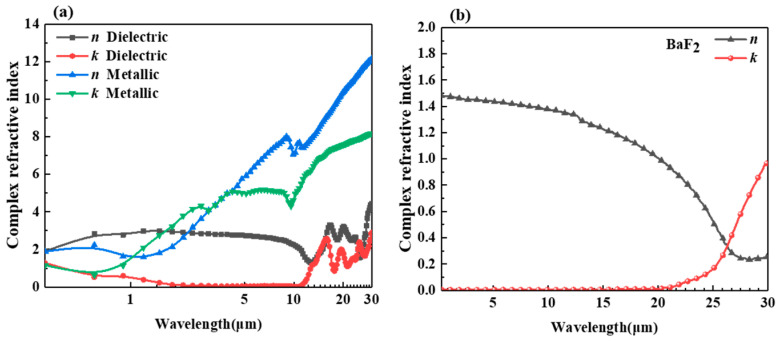
(**a**) Complex refractive index of VO_2_ and (**b**) the complex refractive index of BaF_2_.

In this case, the medium inside the cavity is air, which has a refractive index of 1. Figure 9a show the complex refractive indices of VO_2_ in both its dielectric and metallic states. VO_2_, a typical metal-oxide thermochromic material, has the sharpest reversible phase transition from metallic to insulating states. The phase transition of VO_2_ is influenced by its intrinsic phase transition temperature and its operating temperature. Figure 9b shows the complex refractive index of the aerogel BaF_2_. It is necessary to use an infrared highly transparent material as a matrix base for the smart aerogel. BaF_2_ crystals belong to the cubic crystal system, exhibiting a good resistance to humidity, a high melting point, and a wide range of high light transmission. In the wavelength range of 0.2–10 μm, the highest transmittance can reach more than 90%. BaF_2_ crystal has good optical and mechanical properties. This makes BaF_2_ crystals widely used in infrared and ultraviolet windows and prism substrates. In this case, BaF_2_ is used as an aerogel substrate, and the hollow particles are responsible for regulating the infrared emittance according to the external conditions.

### 4.2. Theoretical Calculations for the Smart Aerogels

The multi-sphere superposition T-matrix is capable of directly calculating the radiative properties of a multilayer spherical structure. This enables the direct computation of the absorption factor, scattering factor, and attenuation factor of the hollow particles, from which the essential data for the Monte Carlo procedure can be derived, such as the refractive index, absorption coefficient, scattering coefficient, asymmetry factor, and the thickness of the thin layer. When calculating the radiative properties of a sparse particle system, in the case of independent scattering, the scattering and absorption coefficients of the system are as follows:

(1) $$C_{sca}=AQ_{sca},C_{abs}=AQ_{abs}$$

(2) $$\beta_{sca}=N_{T}C_{sca},\beta_{abs}=N_{T}C_{abs},\beta_{ext}=\beta_{abs}+\beta_{sca}$$

where A is the projected geometric area of the volume of the sphere; *Q_abs_* is the absorption factor; *Q_sca_* is the absorption factor; $\beta_{sca}$ is the scattering coefficient; and *β_abs_* is the coefficient of absorption. The particle number density *N* is as follows:

(3) $$N_{T}=\frac{3f_{v}}{4\pi r^{3}}$$

where *f*_v_ is the volume fraction of smart-coated doped particles; *r* is the radius of the core–shell particles.

The structure-dependent radiative properties are determined by solving the Radiative Transfer Equation (RTE) to obtain the apparent radiative properties of the smart aerogel. The RTE is employed to describe the incident light transfer within the smart aerogel. Given the low doping concentration and small particle size, the scattering effects during radiative transfer can be considered independent:

(4) $$\frac{dI_{\lambda }(s)}{ds}=-k_{\lambda }I_{\lambda }(s)-\sigma_{\lambda }I_{\lambda }(s)+k_{\lambda }I_{b\lambda }(s)+\frac{\sigma_{\lambda }}{4\pi }\int_{4\pi }I_{\lambda }(s,\vec{\Omega^{\prime }})\Phi_{\lambda }\left(\vec{\Omega^{\prime }},\vec{\Omega }\right)d\Omega^{\prime }$$

where *I*_λ_ and *I*_λ_ are the intensity of spectral radiation along paths in the $\vec{\Omega }$ direction and the intensity of spectral blackbody radiation, respectively.

According to Kirchhoff’s law, under thermal equilibrium, the spectral normal emittance and spectral normal absorptivity of an object are equal at any specific temperature and wavelength. Hence, the spectral normal emittance of a smart aerogel can be expressed as follows:

(5) $$\alpha_{\lambda }=\varepsilon_{\lambda }$$

where $\alpha_{\lambda }$ is the spectral directional absorptance. The radiative transfer equation is solved by the Monte Carlo (MCML) method. In turn, the normal absorptivity of the smart aerogel is calculated. The total normal emittance *ε* is obtained by:

(6) $$\varepsilon =\frac{\int_{0.3}^{20}\varepsilon_{\lambda }I_{B}\left(\lambda ,T\right)d\lambda }{\int_{0.3}^{20}I_{B}\left(\lambda ,T\right)d\lambda }$$

where *I_B_* is defined as the normalized spectral radiance of a blackbody at a temperature of 300 K, normalized by the radiance derived from the Planck function [30]. In the present work, the spectral emittance of smart aerogels based on hollow VO_2_ particles is investigated in the spectral range from 0.3 to 30 μm, corresponding to the main emission bands of a blackbody at 300 K temperature. The tunability Δ*ε* of the emittance is defined as the difference in the total directional emittance between the metallic and dielectric states of the hollow VO_2_ particles in the smart aerogel:

(7) $$\Delta \varepsilon =\varepsilon_{m}-\varepsilon_{d}$$

## Figures and Tables

**Table 1:** Comparison with reported literature on radiation regulation.

Reference	Model	Emissivity Adjustment Rate ∆*ε*	Wavelength Range
Our work	Hollow particle	51.295%	0.3–30 μm
[26]	W/Al Co-doping VO_2_ nanoparticles	48%	8–14 μm
[27]	CaF_2_/VO_2_	36%	4–12.5 μm
[28]	(Filter)/VO_2_/MgF_2_/W	58.2%	8–13 μm
[29]	(Sapphire)/VO_2_/PMMA/Au	60%	8–14 μm
[11]	VO_2_/CaF_2_; VO_2_/ZnS	52.6%; 53.7%	0.3–20 μm

## Data Availability

Data underlying the results presented in this paper are not publicly available at this time but may be obtained from the authors upon reasonable request.
